# Juvenile cystic adenomyoma, a rare diagnostic challenge: Case Reports and literature review

**DOI:** 10.1016/j.xfre.2021.02.002

**Published:** 2021-02-10

**Authors:** Sushila Arya, Heather R. Burks

**Affiliations:** Department of Obstetrics and Gynecology, University of Oklahoma Health Sciences Center, Oklahoma City, Oklahoma

**Keywords:** Accessory uterine cavity masses, cystic myometrial lesions, juvenile adenomyotic cysts, juvenile cystic adenomyoma, Müllerian anomaly

## Abstract

**Objective:**

To report 2 very rare cases of young women who presented with severe dysmenorrhea and a large cystic lesion in the myometrium, which presented a diagnostic dilemma because they were confused with a Müllerian anomaly.

**Design:**

Case reports and a literature review.

**Setting:**

A university-based reproductive endocrinology and infertility clinic in the United States.

**Patient(s):**

An 18- and a 16-year-old nulliparous girl presented with worsening of their longstanding pelvic pain, and imaging study results were suggestive of a Müllerian anomaly.

**Intervention(s):**

Abdominal and pelvic computed tomography, transvaginal ultrasonography, pelvic magnetic resonance imaging, operative laparoscopy, and excision of a juvenile cystic adenomyoma (JCA).

**Main Outcome Measure(s):**

Resolution of the pelvic pain and restoration of normal uterine anatomy after appropriate intervention

**Result(s):**

Restoration of normal uterine anatomy, which was confirmed by 3-dimensional ultrasonography for case 1; however, case 2 still had a small remnant of JCA postoperatively.

**Conclusion(s):**

Clinical and radiologic examinations may not be useful in differentiating a Müllerian anomaly from other rare abnormalities like JCA. When in doubt, laparoscopy can assist in diagnosing and treating the condition.

**Discuss:** You can discuss this article with its authors and other readers at **https://www.fertstertdialog.com/posts/xfre-d-20-00248**

According to Bird et al. (1), adenomyosis is defined as a “benign invasion of the endometrium in the myometrium, producing a diffusely enlarged uterus, which microscopically exhibits ectopic, non-neoplastic, endometrial glands and stroma surrounded by hypertrophic and hyperplastic myometrium.”

Secretory changes and menstrual bleeding within this heterotopic endometrial tissue can result in the formation of tiny hemorrhagic foci (<5 mm) within the myometrium. These foci are typically found in adult parous women of >30 years of age with global/diffuse adenomyosis. These small cysts can be seen as small myometrial cysts using ultrasonography and magnetic resonance imaging (MRI) (2). However, there is a small population of individuals who have larger cysts within the uterine body, termed as “cystic adenomyosis.” These large myometrial cysts in young women have often been described in the literature using various terms, including juvenile cystic adenomyosis (JCA), cystic myometrial lesions, accessory uterine cavity masses, or juvenile adenomyotic cysts. The symptoms presented by these cystic myometrial lesions can be similar to those of obstructive uterine anomalies.

Cystic forms of adenomyosis were first described by Cullen (3) in 1908 as an adenomyomata. Unlike diffuse adenomyosis, this cystic form primarily occurs in adolescents and women younger than 30 years and is not associated with diffuse adenomyosis (4). Takeuchi et al. (5) described JCA based on the following diagnostic criteria: age <30 years; cystic lesion >1 cm in diameter, independent of the uterine cavity, and covered by hypertrophic myometrium, as seen on radiologic images; and association with severe dysmenorrhea (5).

## Materials and methods

A retrospective chart review was performed for both the patients. To identify the previously published cases of JCA, a literature review, with PubMed and Google Scholar, was performed using various combinations of the following search terms: juvenile cystic adenomyoma, juvenile adenomyotic cysts, cystic myometrial lesions, accessory uterine cavity masses (ACUM), adenomyosis, Müllerian anomalies, noncommunicating uterine horn, and cavitated rudimentary uterine horn. Case reports of JCA were included if they met the following criteria: if they met the abovementioned criteria used to include a case as JCA, as described by Takeuchi et al. (5); and adolescent girls and women below 30 years who presented with early-onset severe dysmenorrhea and pelvic pain. We excluded case reports of women who were ≥30 years of age and those who had a prior history of myomectomy or other uterine procedures. Based on the initial list, the reference sections were reviewed, and similar additional articles were identified and included in the review. All cases reported as JCA that met the abovementioned criteria through November 2020 and were published in the English language were included.

All cases reported were histopathologically confirmed JCA that presented with severe primary dysmenorrhea and a large, isolated cystic myometrial lesion revealed by pelvic imaging studies. Consent for this manuscript was obtained from both the patients.

## Case 1

**Figure 1:**
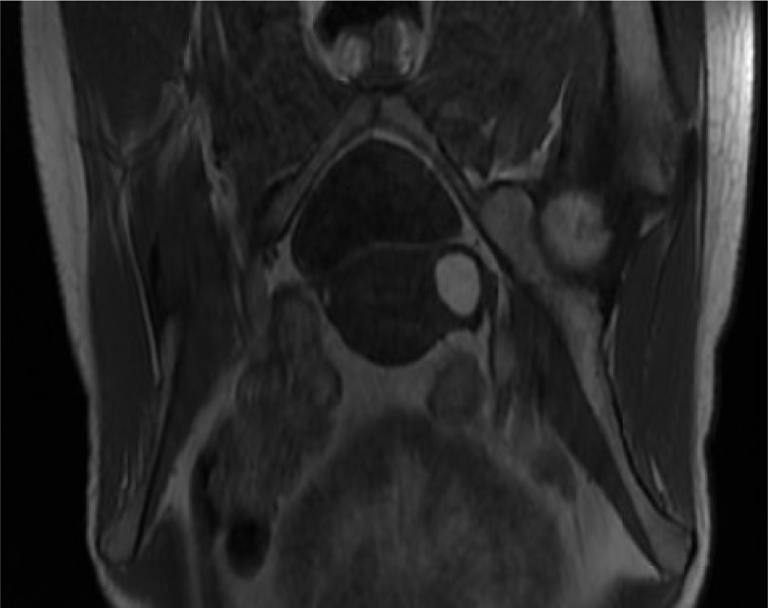
MRI with a coronal T1-weighted image of the pelvis showing a bright T1 well-circumscribed lesion in the left uterine myometrium. MRI = magnetic resonance imaging.

An 18-year-old nulligravida was referred to our clinic for gradually worsening pelvic pain and pelvic imaging result suggestive of a Müllerian anomaly. She started menarche at the age of 14 years. Her periods were always regular at an interval of 28 days and associated with severe dysmenorrhea, with pain starting before the menstrual period and continuing throughout the period. She was not sexually active. The patient underwent abdominal computed tomography (CT) and pelvic scans for the worsening pain, which reported findings consistent with those of bicornuate uterus versus uterine fibroids. She was started on hormonal therapy, with no relief of her symptoms. She subsequently underwent transvaginal ultrasonography, which showed a fluid-filled lesion on the left side of her uterus, concerning for a unicornuate uterus with a noncommunicating horn. Magnetic resonance imaging (performed in February 2016) confirmed these findings (Fig. 1). No urogenital anomalies were noted using MRI. The bilateral adnexae were unremarkable.

The patient was referred to our clinic for worsening pelvic pain and imaging results suggestive of a Müllerian anomaly, with specific indication of a unicornuate uterus with a noncommunicating left horn. Three-dimensional transvaginal ultrasonography was performed at our clinic, showing a normally shaped endometrial cavity, endometrial thickness of 4 mm, and a separate 3 × 3-cm hypoechoic lesion in the left lateral myometrium that appeared to be distinct from the endometrial cavity.

**Figure 2:**
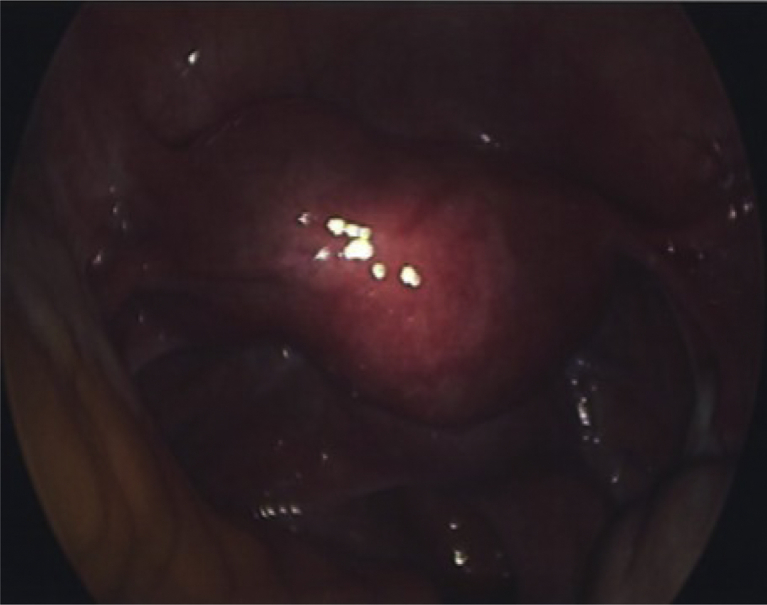
Intraoperative appearance of a cystic adenomyoma located on the left side of the uterus. The bilateral adnexae are unremarkable.

Given the inconclusive findings of the abovementioned imaging studies and the persistent pelvic pain, we recommended diagnostic laparoscopy with possible resection of the cystic mass. The patient gave informed consent before the procedure. The laparoscopy revealed a 3 × 3-cm uterine mass arising from the left lateral uterine wall and extending toward the left side of the broad ligament (Fig. 2). Small foci of endometriotic lesions were noted in the posterior aspect of the cul-de-sac and were fulgurated. Diluted vasopressin was injected into the serosa overlying the mass; an incision in the serosa was made using monopolar scissors. The cyst was ruptured during this process, and a chocolate-brown colored fluid reminiscent of an endometrioma was noted. Suction irrigation was performed to improve visualization, and the inner cavity of the cyst was visually inspected. The appearance of the tissue lining the cyst wall was similar to that of a normal endometrium. The cyst wall was then identified and enucleated, leading to its complete resection using blunt and sharp dissection in the interphase between the myometrium and cystic adenomyoma. The cyst wall was noted to be strongly adherent. The adenomyotic cyst was entirely separated from the endometrial cavity without entering it during cyst removal. The myometrial defect was sutured in 2 layers using a 2-0 vicryl suture. Complete hemostasis was achieved, and the patient’s left fallopian tube was free of injury at the end of the treatment. The surgery was completed with minimal blood loss in approximately 2 hours.

Histopathological examination of the cyst revealed benign smooth muscles with a decidualized endometrium, which was consistent with exogenous hormone therapy—consistent with a noncommunicating uterine horn instead of cystic endometriosis. Complete resolution of the myometrial cystic lesion and resumption of normal anatomy was noted postoperatively using transvaginal ultrasonography. Our patient is 21 years old now, relieved of her pelvic pain after the laparoscopic resection of the cystic adenomyoma. She has been prescribed continuous oral contraceptive pills for menstrual suppression.

## Case 2

A 16-year-old nulligravid woman was referred to our clinic for the management of a cystic uterine lesion associated with acute worsening of chronic dysmenorrhea. She started menarche at the age of 14 years. Her periods were always regular at an interval of 28 days and associated with severe dysmenorrhea. She was prescribed oral contraceptive pills for dysmenorrhea, with partial relief. Before her referral to our clinic, she presented to the emergency room twice for worsening of pain in the lower quadrant. She underwent CT and ultrasound imaging, which revealed a cyst in the right lateral myometrium, consistent in its echotexture with that of an endometrioma. A CT of the abdomen and pelvis revealed a 4.7 × 3.8-cm subserosal cystic mass on the right side of the uterus, which appeared to be discrete from the endometrium. Pelvic ultrasonography was performed on the same day, which revealed a cystic lesion measuring 5.1 × 3.6 × 4.8 cm, with an echotexture consistent with that of an endometrioma, either within the cornua or in the fallopian tube; however, the exact location was difficult to identify using ultrasound.

**Figure 3:**
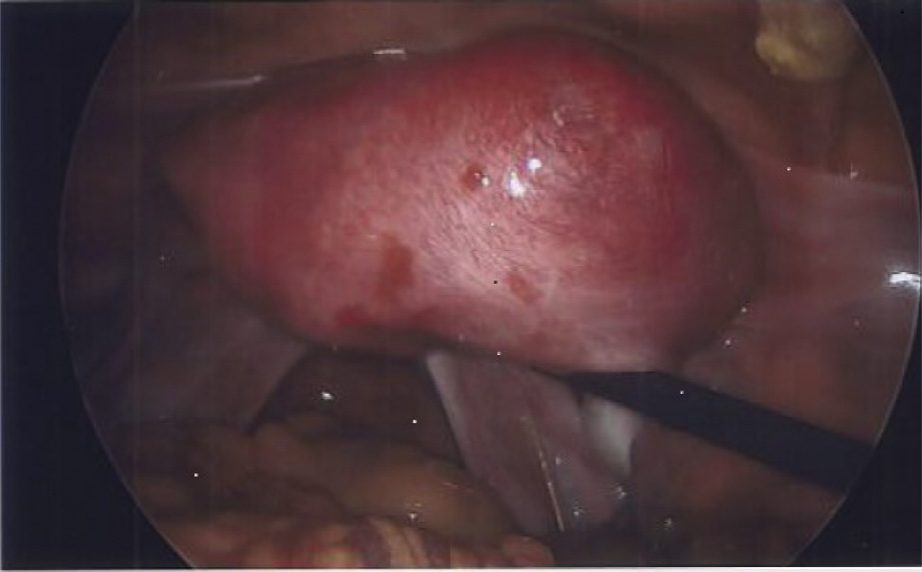
Intraoperative appearance of a cystic adenomyoma located on the right side of the uterus. The bilateral adnexae are unremarkable.

The patient underwent diagnostic laparoscopy, which confirmed a bulge arising from the right uterine cornua and the presence of a cystic mass, indicative of a Müllerian anomaly. Appendectomy was concurrently performed because of a rigid and hyperemic appearance of the appendix, and minimal endometriosis was fulgurated. She was referred to us for further care and worsening of the pelvic pain after she underwent the abovementioned laparoscopy. We performed laparoscopic excision of the uterine cyst following a procedure similar to that used for case 1. The intraoperative findings (Fig. 3) were a 5-cm bulging mass arising from the right cornual region and unremarkable bilateral adnexae. The final pathology report confirmed JCA. The patient experienced proper recovery and pain relief immediately after the laparoscopic resection of the adenomyoma. During follow-up ultrasonography, a cystic area in the left cornual region was noted, concerning to be a remnant of JCA. She now requires continuous oral contraceptive pills for the pelvic pain and suppression of endometriosis.

Table 1 summarizes a selected review of the literature related to JCA. In a report by Takeuchi et al. (5), the timing of the onset of severe dysmenorrhea was a mean of 6.6 years after menarche, ranging from 1 to 13 years later. These lesions are commonly 3–4 cm in size, although it may vary from 2 to 8 cm. The most common location is the lateral uterine wall under the round ligament insertion, without interruption of the fallopian tubes, and the uterine cavity is typically unaffected. Coexisting diffuse adenomyosis was identified using MRI in 4 cases of JCA. Five patients in the case series by Takeuchi et al. (5) had peritoneal endometriosis, similar to our patients, and 1 patient had ovarian endometrioma. The effect of the diagnosis of JCA and surgical resection of these lesions on future reproductive potential has not been well described. Takeuchi et al. (5) reported that 2 of 3 patients who desired pregnancy had successful live births after resection.

**Table 1:** Review of literature related to juvenile cystic adenomyoma.

Author, year	No. of patients	Age at the time of intervention^b^	Size of lesion (mm)	Laterality	Coexisting adenomyosis	Imaging	Medical management	Surgery	Outcome
Tamura et al., 1996 (12)	1	4 y after menarche	30	NA	No	US MRI	NA	LT	Complete
Potter and Schenken, 1998 (13)	1	15 y	40	NA	Yes	US HSG	Incomplete	LT	Complete
Fisseha et al., 2006 (14)	1	13 y	30	Left	No	US MRI	Good	Not attempted	Stable on meds
Takeda et al., 2007 (6)	2	At menarche	3026	NA	No Yes	US MRI HSG	Incomplete	LPS	Complete
Dogan et al., 2008 (15)	1	19 y	35	Left	NA	US MRI		LT	Complete
Ho et al., 2008 (16)	1	15 y	NA (large)	Right	No	US CT MRI	NA	LT	NA
Ball et al., 2009 (17)	1	4 y after menarche	20		No	US	Incomplete	LPS	Complete
Takeuchi et al., 2010 (5)	9	25.2 y^a^	31^a^	NA	Yes, in all	US MRI HSG	Incomplete	LPS	Complete
Akar et al., 2010 (18)	1	15 y	47	Right	NA	USG CT	Incomplete	RAL	NA
Chun et al., 2011 (19)	1	19 y	30	Left	No	MRI	Incomplete	LPS	Complete
Kriplani et al., 2011 (20)	4	20 y^a^	NA	NA	NA	NA	Incomplete	LPS	Complete
Jain et al., 2012 (21)	2	19 y 22 y	NA	Left Right	No	US MRI	Incomplete	LPS	Complete
Branquinho et al., 2012 (22)	1	17 y	33 mm	Right	No	US MRI	Complete	None	Complete
Cucinella et al., 2013 (23)	1	25 y	45	NA	NA	US MRI	Incomplete	LPS	Complete
Kumakiri et al., 2013 (24)	1	20 y	30	NA	NA	US	NA	LPS	Complete
Pontrelli et al., 2015 (25)	1	27 y	80	Posterior wall	NA	US MRI HSC	Incomplete	HSC	Complete
Dadhwal et al., 2017 (26)	2	23 y 16 y	40^a^	Right Left	NA	US MRI	Incomplete	LT, LPS	Complete
Peters et al., 2018 (10)	1	19 y	30	Left	NA	US MRI	Not attempted	RAL	NA
Protopapas et al., 2020 (27)	1	14 y	38	Left	NA	MRI	Not attempted	LPS	Complete
Wilcox et al., 2020 (28)	2	18 y, 18 y	23, 36	Left	No	3D US MRI	LNG IUD CHC	HSC LPS	Complete
Kiyak et al., 2020 (8)	1	27 y	42	Right	No	US	None	LPS	Complete

US = ultrasonography; CT = computed tomography; MRI = magnetic resonance imaging; HSG = hysterosalpingogram; HSC = hysteroscopy; NA = information not available; LT = laparotomy; LPS = laparoscopy; RAL = robotic-assisted laparoscopy. LNG IUD = levonorgestrol intrauterine device; CHC = combined hormonal contraceptive.

a Values are represented as means.

b Some case studies did not mention the age at which the patient received an intervention but mentioned the age at the time of menarche.

## Discussion

Large myometrial cysts in young women are very infrequent. The most common presenting symptoms are severe dysmenorrhea and pelvic pain, with inadequate relief when typical medical management strategies such as nonsteroidal anti-inflammatory drugs and combined hormonal contraceptives meant for ovulation suppression are used.

The etiology of juvenile cystic adenomyosis is not clear, although various investigators have proposed different theories in an attempt to identify the origin of these rare uterine abnormalities. Based on early-onset severe dysmenorrhea soon after menarche and the typical uterus-like organization of JCA, Takeda et al. (6) suggested that JCA results from developmental defects of the Müllerian ducts. Although JCA is associated with a normal uterine cavity, unlike other Müllerian anomalies, Acién et al. (7) hypothesized that cavitated uterine lesions in premenopausal women represent a previously unrecognized Müllerian anomaly that results from gubernaculum dysfunction, leading to duplication or persistence of paramesonephric tissue. In contrast, Takeuchi et al. (5) consider it a cystic variant of adenomyosis. These 2 entities have been considered to have the same underlying process (7) and similar clinical presentation or different processes but with the main clinical difference being the presence of dense adenomyosis in JCA (8).

Although ultrasound remains the most common imaging modality for evaluation, MRI has also been shown to be useful in the evaluation of these cystic structures when they are not clearly defined using ultrasound. Using 3-dimensional transvaginal ultrasonography, a cystic adenomyoma can be visualized as a round cystic mass filled with hypoechoic content, separate from the normal uterine cavity, as seen in our first case, typically in the cornual region. On MRI images, cystic adenomyosis appears to be a well-circumscribed cystic lesion filled with hemorrhagic fluid in different stages of organization within the myometrium (5).

Menstrual suppression using continuous oral contraceptive pills, gonadotropin-releasing hormone analogs, and nonsteroidal anti-inflammatory drugs may provide temporary and partial pain relief in JCA (5, 9), but laparoscopic resection remains the most effective treatment (5). The recurrence of JCA has not been reported yet. Majority of the reported cases successfully completed the resections of these cystic adenomyosis lesions laparoscopically, with complete resolution of pelvic pain. Peter et al. (10) published a narrated video describing a fertility-sparing, minimally invasive, surgical technique to enucleate and resect cystic adenomyosis lesions. Magnetic resonance imaging findings may prove useful in selecting the right approach to locate and enucleate the lesion.

Efforts to unfold the etiology of juvenile cystic adenomyosis can continue with better awareness of the condition and imaging modalities. Based on our comprehensive review of previous JCA cases (Table 1), significant delay in diagnosis and treatment is evident. A Müllerian anomaly, a degenerating leiomyoma, and an ovarian endometrioma adjacent to the uterus are also part of a reasonable differential diagnosis of myometrial cystic lesions (11). However, more cases need to be investigated to develop a uniform effective protocol for diagnosing and treating a rare disorder like JCA.

## Conclusion

Based on our experience and the literature review of JCA cases, we suggest that pelvic ultrasonography for young women with severe dysmenorrhea, performed transvaginally when appropriate and transabdominally in other patients, be considered the first diagnostic modality. When an initial imaging result does not lead to a clear diagnosis, MRI should be performed. Diagnostic laparoscopy should be offered when the diagnosis remains unclear after MRI or when dysmenorrhea is unresponsive to medical management. This also provides an opportunity for surgical management with mass resection, which might effectively help relieve symptoms.
